# Additive Manufacturing of Miniaturized Peak Temperature Monitors for In-Pile Applications

**DOI:** 10.3390/s21227688

**Published:** 2021-11-19

**Authors:** Kiyo T. Fujimoto, Lance A. Hone, Kory D. Manning, Robert D. Seifert, Kurt L. Davis, James N. Milloway, Richard S. Skifton, Yaqiao Wu, Malwina Wilding, David Estrada

**Affiliations:** 1 Idaho National Laboratory, 1955 N. Fremont Ave., Idaho Falls, ID 83415, USA; kiyofujimoto@u.boisestate.edu (K.T.F.); lance.hone@inl.gov (L.A.H.); kory.manning@inl.gov (K.D.M.); Robert.Seifert@inl.gov (R.D.S.); Kurt.Davis@inl.gov (K.L.D.); James.Milloway@inl.gov (J.N.M.); Richard.Skifton@inl.gov (R.S.S.); Malwina.wilding@inl.gov (M.W.); 2 Center for Advanced Energy Studies and Micron School of Materials Science and Engineering, Boise State University, 1910 University Dr., Boise, ID 83725, USA; yaqiaoWu@boisestate.edu

**Keywords:** aerosol jet printing, in-pile sensors, nuclear energy, additive manufacturing, advanced manufacturing, peak temperature sensors

## Abstract

Passive monitoring techniques have been used for peak temperature measurements during irradiation tests by exploiting the melting point of well-characterized materials. Recent efforts to expand the capabilities of such peak temperature detection instrumentation include the development and testing of additively manufactured (AM) melt wires. In an effort to demonstrate and benchmark the performance and reliability of AM melt wires, we conducted a study to compare prototypical standard melt wires to an AM melt wire capsule, composed of printed aluminum, zinc, and tin melt wires. The lowest melting-point material used was Sn, with a melting point of approximately 230 °C, Zn melts at approximately 420 °C, and the high melting-point material was aluminum, with an approximate melting point of 660 °C. Through differential scanning calorimetry and furnace testing we show that the performance of our AM melt wire capsule was consistent with that of the standard melt-wire capsule, highlighting a path towards miniaturized peak-temperature sensors for in-pile sensor applications.

## 1. Introduction

For over a century burning fossil fuels has provided most of the energy required to heat our homes, power industry, drive our cars, and light up our cities. Within the United States alone, 85% of total energy comes from oil, coal, and natural gas [1]. While fossil fuel based energy consumption continues to increase alongside the world’s population, growing concern over limited fossil fuel supplies and the accumulating greenhouse gases in the Earth’s atmosphere have triggered a transition from using fossil fuels as our main energy source to those that are more sustainable and environmentally responsible [2]. Of the clean energy sources available there is only one zero-carbon technology capable of meeting most, if not all, of the energy demands of a modern society, and that is nuclear power. Severe reactor accidents such as Fukushima Daiichi Nuclear Power Plant (2011) have emphasized the need to enhance accident tolerance in nuclear plants and prompted developments in advanced fuels and more robust structural materials capable of enhancing safety and efficiency in operations [3]. Nuclear energy has had a declining share in global electricity markets., and if nuclear energy is to realize a global impact in clean energy production and reduced carbon emissions, innovations in nuclear technology are needed to extend the life of current light-water reactors and expedite the development of generation four nuclear reactors [4,5,6].

On a fundamental level, safety in nuclear reactor operations requires an in-depth understanding of how advanced nuclear fuels and structural materials behave within a reactor environment [7]. The development, demonstration and qualification of advanced reactor materials is expected to facilitate an accelerated deployment of advanced reactor technologies, and this requires a greater understanding of the irradiation effects on fuels and material behaviors [8]. Furthermore, this understanding is critical for the assessment of potential materials for any nuclear reactor concept to ensure safety, reliability and efficiency in operations. This understanding is typically acquired with the use in-pile (i.e., in-core or in-reactor) sensors deployed as part of experiments in Materials Test Reactors (MTRs). MTRs such as the Advanced Test Reactor (ATR) and the Transient Reactor Test Facility at the Idaho National Laboratory use specialized irradiation capsules equipped with in-pile instrumentation for targeted property measurements within the extreme environment of a nuclear reactor core [9,10,11]. Current in-pile instrumentation efforts look to assess, verify, and increase the precision of measurements under irradiation with the development of advanced sensors capable of monitoring temperature, physiochemical conditions, neutron flux/dose, pressure, and multi-physics field properties. To maximize time, resources, and data collection, advanced and miniaturized instrumentation is needed to accurately measure such properties in the extreme and complex environment of nuclear test reactors.

A key parameter during irradiation tests is temperature. Temperature monitoring is currently achieved with both passive- and active-monitoring techniques [12]. Active-monitoring techniques, such as thermocouples, provide real-time data and are typically expensive because they require the implementation of instrumentation leads. On the other hand, passive-monitoring techniques are typically used in static irradiation capsules, and can provide an insight toward peak temperatures [13]. For those instances where irradiation tests are seeking a less-expensive measurement method and/or the experiment requires instrumentation without leads such as static capsule experiments, passive techniques are the preferred method for temperature monitoring [10].

Melt wires are a passive monitoring technique that enables identification of the peak temperature achieved during an irradiation test [14]. This method involves placing wires of a known composition and well-characterized melting temperature within an experimental test capsule designed for materials testing. The peak test temperature is then inferred during post-test examination or post-irradiation examination (PIE) where the melt wires are inspected for visual signs of melting. If the material shows signs of melting it can then be reasoned that the peak temperature during testing exceeded the melting point of melt wire material. On the other hand, it is determined that the peak test temperature remained below the melting point of the wire material if the wire does not show signs of melting. Preferably, materials chosen for melt wires have a low neutron-absorption cross-section while exhibiting distinct and reproducible melting behavior when they have been exposed to temperatures beyond their respective melting point.

Current state-of-the art passive peak temperature sensors used within MTRs are melt wires, and within this work will be referred to as classical melt wires. These melt wires have been matured through a meticulous material selection process and the development of validation procedures to confirm their temperature dependent properties. The process for classical melt-wire fabrication includes the ability to encapsulate multiple wire materials into one small-diameter unit which contains the samples under inert atmosphere [15]. The library of qualified materials for melt-wire selection contains more than 40 useful materials with a detection range between 29.73 °C and 1535 °C [15]. Wire materials are chosen based on expected irradiation test temperatures and required temperature measurement resolution [14]. While classical melt wires are commonly used in test-reactor experiments, such as those conducted in the ATR, some test designs have limited space due to predesigned capsules that may only be a couple of millimeters in diameter. As multiple specimens are contained within the capsules at once, this can leave little or no space for passive and/or active instrumentation.

The production of miniature, robust peak temperature sensors is made possible through additive manufacturing (AM) techniques such as aerosol-jet printing (AJP), ink-jet printing (IJP), and micro-dispense printing (MDP). The incorporation of these AM techniques in the design of in-pile instrumentation enables the development of advanced sensors for MTRs with features as small as 10 μm, which is advantageous for device miniaturization, especially when considering that traditional melt-wire capsules typically require a wire length of approximately 2 mm [16,17]. Currently, novel technologies such as AJP are being explored for the development of unique sensors that are otherwise unobtainable with conventional fabrication processes [13,18,19,20,21].

The ability to directly deposit functional materials onto a wide range of substrates using AM techniques makes it possible to expand the encapsulation design and methods used for melt wire fabrication. AM facilitates the miniaturization of the entire melt wire package to accommodate more irradiation experiments. While the actual melt wires may be further miniaturized with currently available microfabrication techniques, the need to miniaturize the entire melt wire package is severely limited with the use of classical fabrication methods and materials. This is because classical melt wires are sealed within a quartz capsule to suspend and separate melt wires under inert atmosphere. The use of quartz as the encapsulation material is the limiting factor in further miniaturizing the melt wire package, as glass-blowing techniques can only allow for a certain degree of miniaturization before the integrity of the melt wire package is compromised. A solution to this is to transition the encapsulation material away from quartz to metals, which can be easily machined and welded.

Classical machining techniques, such as milling, can produce small components such as a very small container with a corresponding lid to contain printed melt wires sealed under inert atmosphere via laser welding or other metal joining techniques. Using AM techniques wires could be easily printed on the base of the container, and through thoughtful design of the container and lid this would provide a way seal to the melt wires within an inert atmosphere, which is critical for melt wire performance. Fabricating a melt wire packages in this way is only afforded by utilizing AM methods.

Previous work has successfully demonstrated the ability to fabricate low-cost and compact melt wire chips by printing silver melt wires within a 2 mm 316 L chip with a first iteration of an encapsulation design for AM melt wires [22]. To expand on that work, the study reported here aims to demonstrate and benchmark the performance and reliability of AM melt-wire materials when compared to their classical counterparts. This work demonstrates the feasibility of AM techniques for the fabrication of advanced nuclear in-pile passive temperature sensors. The performance of prototypical classical melt wires serves as a benchmark to compare the performance of AM melt wire capsules containing aluminum (Al), zinc (Zn), and tin (Sn) AJP deposited melt wires. To minimize the number of variables introduced during benchmark testing, the encapsulation method for both the traditional and AM melt wires was based on classical fabrication techniques that do not allow for overall device miniaturization. Second, significant design improvements to the encapsulation design for AM melt wires from the first iteration were introduced with a second iteration to allow for an inert atmosphere within the capsule, which is critical for melt wire performance, especially when melt wire oxidation is of concern. The melting point of each of these materials encompasses the operating temperatures of a wide range of different reactor systems [23,24]. For these studies, the low melting-point material used was Sn, with a melting point of approximately 230 °C, Zn melts at approximately 420 °C, and the high melting-point material was Al, with an approximate melting point of 660 °C.

## 2. Materials and Methods

### 2.1. Materials

The incorporation of advanced fabrication methods of sensors for melt wires requires the development of ink materials containing melt-wire materials of interest while demonstrating compatibility with additive-printing technologies. For this study, AJP-compatible inks were synthesized that contained the melt wire materials of interest. Melt wire ink feedstock materials were purchased as nanopowders. Aluminum nanopowder (99.9%, 800 nm, 1–2% PVP-coated), zinc nanopowder (99.9%, 95–105 nm, 1–2% PVP-coated) and Sn nanopowder (99.9%, 60–80 nm, 1–2% PVP-coated) were all acquired commercially from US Research Nanomaterials (Houston, TX, USA). Nanopowder dispersion was achieved with the use of USP grade 200-proof ethanol (Fisher Scientific, Waltham, MA, USA), high purity ethylene glycol (VWR Life Science, Radnor, PA, USA), and BYK-156 (BYK USA Inc., Wallingford, CT, USA). All chemicals and reagents were used as received, without further purification or modification.

### 2.2. Methods

#### 2.2.1. Ink Synthesis

First, a stock solution was created to act as the dispersion medium for nanopowders; it was composed of 0.1 g of BYK-156 added to a 1:3 solution of ethanol to ethylene glycol, which was allowed to stir for 1 h. Three different AJP inks were created from each individual nanopowder. For the optimization process, inks, having a nanopowder loading of 60 wt%, were initially produced using a high shear mixer (5000 RPM for 15 min; L5M-A equipped with a 5/8 in. microtubular mixing unit, Silverson, East Longmeadow, MA, USA). To benchmark the performance of the initial ink composition, AJP was used and, depending on the consistency of the output, additional solvent was added. If necessary, the dispersion medium was added in 5 mL increments and then subjected to high-shear mixing. This process continued until the desired output and ink performance were achieved. Final concentrations for each of the inks were as follows: 45 wt%, 60 wt%, and 40 wt% for tin, aluminum, and zinc, respectively.

#### 2.2.2. Powder Feedstock Particle Size and Composition Analysis

Powders were characterized with transmission electron microscopy (TEM) for particle size analysis, X-ray diffraction (XRD) analysis for identifying powder composition and differential scanning calorimetry (DSC) to identify the melting point of the powder feedstock. Particle size analysis was performed with images obtained with a FEI Tecnai F3-FEG STwin TEM with EDS using a single tilt holder and a Formvar/Carbon 200 mesh copper grid (Ted Pella, Inc., Redding, CA, USA). ImageJ software was used to evaluate the average particle size for each powder feedstock. Confirmation of the feedstock powder composition was accomplished with XRD analysis using a Rigaku Smart Lab operating with a parallel beam geometry in combination with PDXL software.

#### 2.2.3. Differential Scanning Calorimetry (DSC)

Stock materials and AJP inks used to fabricate the standard and AM melt wires, respectively, were subjected to DSC (Netzsch DSC 404 C Pegasus, Burlington, MA, USA) to evaluate the melting temperature of each material to validate the melting point of the actual stock material used, rather than relying on standard data. Alumina crucibles were used for determining the melting phase transitions of experimental samples. A four-step temperature program consisting of: (1) a ramp of 5 °C/min until a targeted temperature of 50 °C below the expected melting point of the material was reached; (2) a reduction of the ramp rate to 1 °C/min until a target temperature of 50 °C above the expected melting point was reached; (3) cooling the sample to the targeted temperature of 50 °C below the expected melting point of the material and holding for one-hour dwell to ensure that the DSC had truly come down to the targeted temperature; (4) repeating this process three more times This temperature profile was repeated for a total of four times, and the onset temperature was used as expected melting point for the printed melt wires. This analysis was completed using the last three runs with Proteus^®^ software (Netzsch).

#### 2.2.4. Additive Manufacturing of Melt Wires

AM of melt wires was accomplished with the pneumatic atomizer (PA) of an Optomec Aerosol Jet 200, equipped with a 200 μm nozzle. The bubbler solvent add-back system, employing a 1:1 ratio of ethylene glycol to ethanol, was used to minimize the effects of solvent loss during atomization. During printing, the ink was held at 35 °C to optimize ink atomization. The tool platen temperature (65 °C), pneumatic atomizer (600 CCM), virtual impactor (500 CCM), and sheath gas (50 CCM) flow rates were optimized to ensure that the line widths and material deposition of functional materials were adequate to obtain the desired device dimensions. SiO_2_ on Si was utilized as the substrate for the printed melt wires. Prior to printing, the substrates were triple rinsed (acetone, methanol and nanopure water) within an ultrasonic bath to clean the substrate surface. After each subsequent cleaning, nitrogen gas was used to dry the substrate.

**Figure 1:**
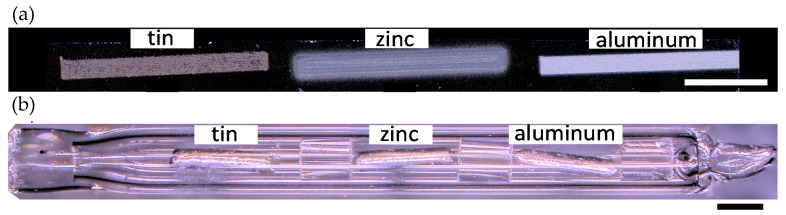
Additively manufactured and classical melt wire capsules. Prototypes of (**a**) AM melt wires of aluminum, zinc and tin fabricated by aerosol jet printing on an SiO_2_/Si substrate, and (**b**) a classical melt wire capsule containing aluminum, zinc and tin. For testing, both AM and classical melt wires were contained within quartz capsules with a helium atmosphere at ambient pressure. Scale bars represent 1 mm.

The printed melt wires were sintered within a tube furnace (MTI GSL-11X-NT) at a temperature of 200 °C for 1 h in a reducing atmosphere (2% H_2_ with nitrogen balance, 50 mL/min) to minimize oxidation while sintering and to remove any residual solvent. The aerosol jet printed melt wires of tin, zinc and aluminum, shown in Figure 1a., were fabricated to have a geometry of 0.25 mm (W) × 20 mm (L) in order to compare their performance to that of a standard melt wire. The wires were sealed within a quartz tube with a helium atmosphere. The capsule was verified as a sealed instrument prior to furnace testing. A similar capsule containing classical melt wires is shown in Figure 1b. For the second part of this study, improvements to the AM melt wire encapsulation design from the first iteration were made. First attempts at miniaturizing melt wire packaging included a 2 mm diameter and 0.5 mm thick Stainless Steel 316 (SS316) disc. It was milled to create a dimple or a pocket and melt wire materials that were printed within the dimple. Next, a blank SS316 disc was placed on the printed melt wire chip, and laser micro-welded to seal and encapsulate the printed materials within SS316 [22,25].

**Figure 2:**
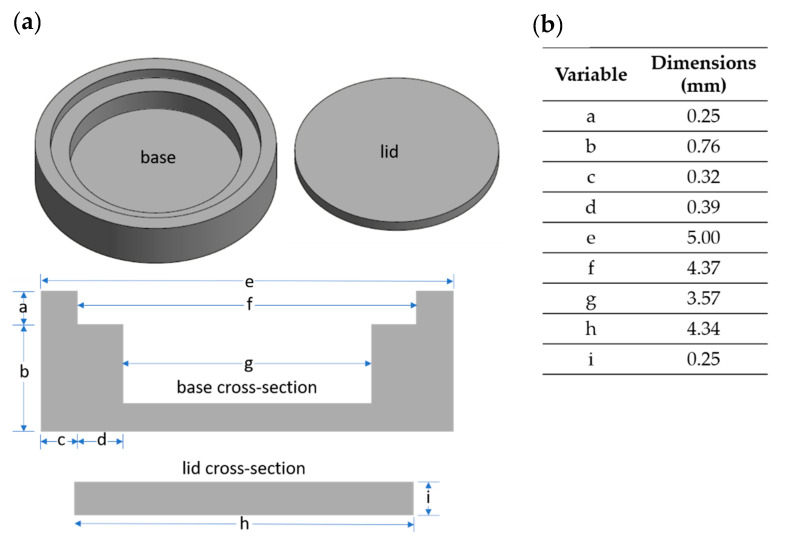
New AM melt wire encapsulation. (**a**) A schematic of the new encapsulation design with base and lid cross-section shown, and (**b**) dimensions of the base and lid.

This work improves on the encapsulation design by addressing two critical features, material thickness and the environment within the capsule as shown in Figure 2. First, the thickness of the encapsulation material was significantly reduced to a side wall thickness of 0.6 mm and a floor thickness of 0.2 mm. Starting with a 5 mm SS316 rod (McMaster-Carr), an initial cut was made using a 3.57 mm end mill to a depth of 0.76 mm followed by a second cut to create a shoulder for the lid to sit recessed within the encapsulation. The second cut, which overlaid the first cut, made with a 4.35 mm end mill to a depth of 0.25 mm. Then, a cap (4.4 mm × 0.254 mm) was milled to fit within the second cut. Dimensions and a schematic of this design can be found in Figure 2.

**Figure 3:**
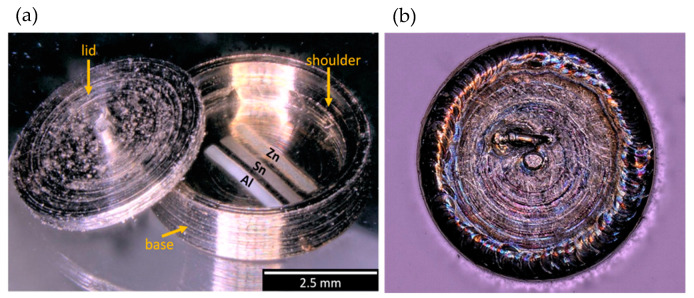
Inert atmosphere encapsulation design for AM melt wires. (**a**) Melt wire container ready to be sealed with AM melt wires on the base of the encapsulation package, and (**b**) a top-view of the sealed capsule.

For this sealing process, changes included the design of a top lid to be attached using a precision laser welder. To aid in the laser welding process, a shoulder was built into the wall of the base to allow the lid to sit recessed in the container such that the lid and base provided a flush surface to weld on (Figure 3a). Laser welding was accomplished with a LaserStar Fiberstar Workstation 7600, and seal welds were completed using 180 W with specific settings at 2.6 J, a pulse width of 16 ms, and a beam diameter step of 5.

On the center of the lid, a small extrusion was added as a feature to assist with handling. To create an inert atmosphere within the encapsulation, a small weep hole was created in the lid (approximately 250 µm) prior to attaching the lid to the base, and a weld was created around the entire lid in air. While laser welding the lid, applying too much energy during the sealing process initially melted the printed melt wires with lower melting points. To prevent premature melting, it was necessary to use a copper cradle fit to press against the melt wire container base to serve as a heat sink during the laser welding process. To ensure the melting point of the melt wires was not exceeded during welding, temperature was monitored on the prototypes with a thermoelement (type K) directly attached to the substrate where the printed melt wires were located. Laser welds were then performed using the energy necessary to close the lid. After a few modifications to ensure a tight fit from the copper to the container, temperatures could be limited to a maximum of 135 °C, ensuring no premature melting of the melt wire materials.

Next, an inert atmosphere was introduced into the sealed container by suspending it within a vacuum system to purge all air and was backfilled with high-purity helium. The final seal was completed by shooting the laser through a quartz window while the melt wire container was in view within the vacuum system. To ensure that an inert atmosphere was maintained within the container, the sealed piece (Figure 3b) was subjected to a helium leak check to confirm that a true seal exists.

#### 2.2.5. Classical Melt Wire Fabrication

Classical melt-wire fabrication involves a series of activities that can be completed repeatedly to ensure reliable and consistent results. For this study, wires of aluminum (99.999% metals basis, with 0.5 mm dia., Puratronic), zinc (99.994% metals basis, with 0.5 mm dia., Puratronic) and tin (99.9% metals basis, with 0.5 mm dia., Leico Industries, Inc., Lyndhurst, NJ, USA) were used to fabricate wires 2 mm in length (Figure 1b). Particular attention was given to cleaning each piece to minimize the possibility of impurities within the final product. The wires were then sealed within a quartz tube with quartz spacers placed between separate materials under vacuum in a helium atmosphere. Quartz spacers were placed between them to separate the materials during the experiment. The capsule was verified as a sealed instrument and furnace tests were conducted to examine performance, observe material interactions between melt wires or the quartz containment tube, and to provide the insight required to visually discern melting after heating in the quartz encapsulated tubes.

#### 2.2.6. Furnace Testing

For both melt-wire types and for both studies the tube furnace (Lindberg clam shell tube furnace max 1200 °C) was brought up to the target temperature, and the melt wires were inserted so that they were positioned vertically during testing. To evaluate performance, each melt capsule was tested at a temperature slightly below and above the materials melting point, as determined by DSC. Tin was evaluated at 215 and 245 °C, zinc at 405 and 435 °C, and aluminum at 640 and 675 °C.

#### 2.2.7. X-ray Computed Tomography

A General Electric (GE) Phoenix vǀtomeǀx nXCT system was used to collect X-ray radiographs and perform micro-computed tomography (XCT). The X-ray generator used was a GE 180 kV nano-focus X-ray tube (Model # XS180NF) with a 5-micron spot size. A GE Dynamic 41–100, flat-panel X-ray detector was used. The detector has a 100 μm pixel pitch and an active area of 410 mm × 410 mm. CT was possible through a precision rotation stage located between the source and detector.

The settings for the X-ray generator were 75 kV and 225 μA, with an aluminum filter that was 0.5 mm thick. The magnification was 24.99× which resulted in a 4.000176 µm Voxel size. A total of 2200 projections were collected resulting in a 0.18° rotation per projection. Eight exposures were averaged for each projection with a 500.031 ms timing. GE’s proprietary reconstruction software, Phoenix Datos, was used for tomographic reconstruction.

Evaluation of melt wires is accomplished by obtaining initial XCT images of a sealed melt wire assembly before the experiment to serve as a reference point. Then, after the experiment a new XCT image is obtained and compared against the reference image to determine if any changes had occurred. Part of the fabrication process is to test prototypes of each material using the same imaging techniques used for PIE to identify any characteristics that indicate melting has occurred.

## 3. Results

### 3.1. Powder Feedstock Characterization

**Figure 4:**
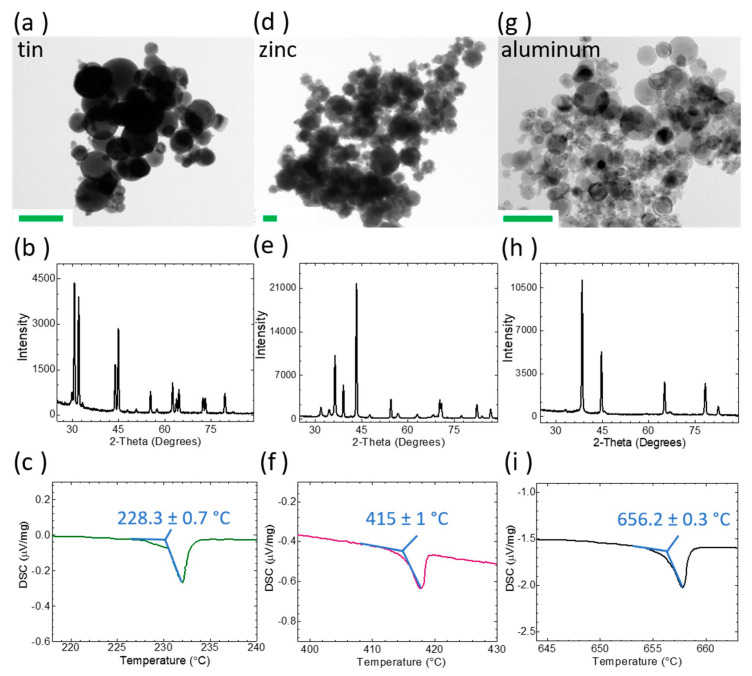
Characterization of melt wire feedstock. Melt wire feedstock powders of (**a**–**c**) tin, (**d**–**f**) zinc and (**g**–**i**) aluminum were characterized for particle size with transmission electron microscopy (TEM) (**a**,**d**,**g**), composition with X-ray diffraction (XRD) (**b**,**e**,**h**), and melting point with differential scanning calorimetry (DSC) (**c**,**f**,**i**). Scale bars are 200 nm.

Once melt wire materials are selected, they are ordered from reliable vendors that provide a certification of purity that remains traceable throughout the fabrication process. For AM melt wires, characterization of the powder feedstock is critical for assessing compatibility with the selected additive technology and identifying material composition. The average particle size was evaluated with TEM imaging (Figure 4a,d,g) for tin, zinc and aluminum powder feedstocks was determined to be 92 ± 50 nm, 230 ± 100 nm and 76 ± 27 nm, respectively. Powder composition was confirmed with XRD (Figure 4b,e,h), and it was anticipated that the melting point for each powder feedstock would be in good agreement with standard values. Although the powder feedstock is purchased from a reliable vendor and composition is confirmed with XRD, when the physical material is received, it is verified by empirically evaluating its melting point using DSC (Figure 4c,f,i) to detect heat absorption of the sample as it undergoes the endothermic phase transition from solid to liquid. The expected melting point derived from DSC analysis for each melt wire fabricated with AJP inks of tin, zinc or aluminum nanopowders are 228.3 ± 0.7 °C, 415 ± 1 °C, and 656.5 ± 0.3 °C, respectively.

### 3.2. AM and Standard Melt Wire Performance Evaluation

Classical and AM melt wires, encapsulated within quartz tubing (Figure 1a,b), were then subjected to furnace testing within a helium atmosphere to examine the performance, observe material interactions, and explore any interactions with the quartz encapsulation tube or SiO_2_/Si substrate of the melt wires. Additionally, this evaluation method provides an understanding for any morphology changes that would indicate that melting has occurred.

**Figure 5:**
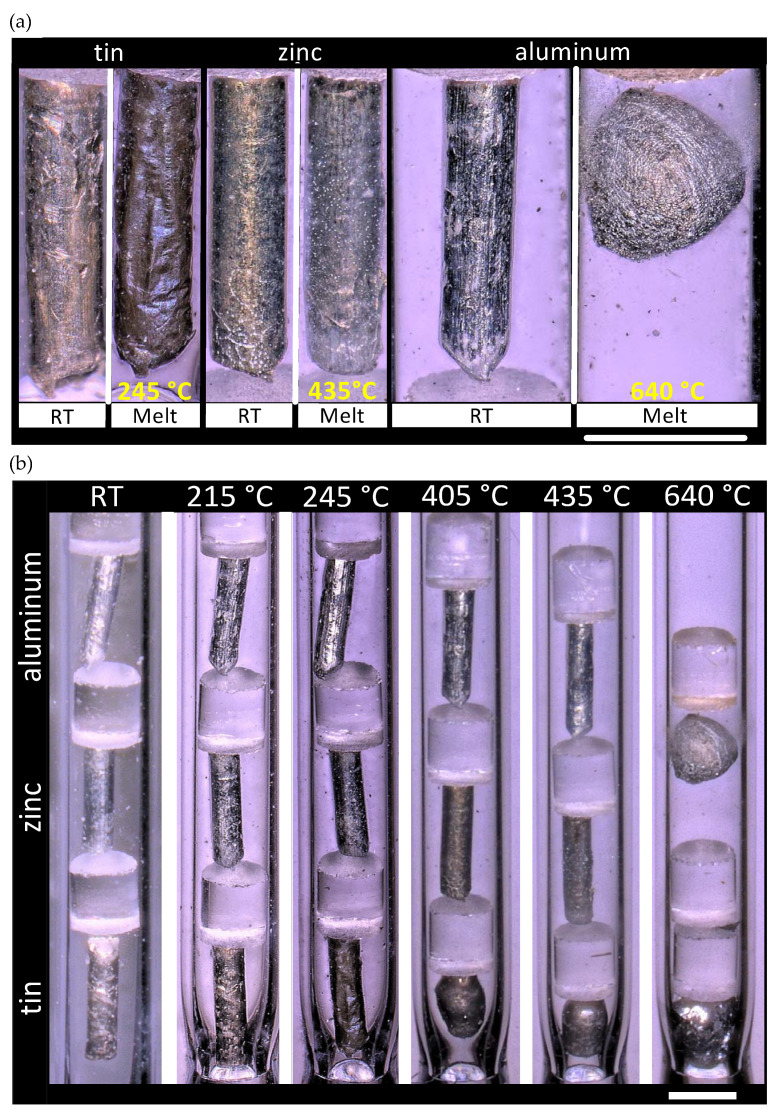
Standard melt wire capsule furnace testing: magnified view of the visual temperature response indicating melting of (**a**) individual melt wires of Sn, Zn and Al, and (**b**) of the full capsule. Scale bars represent 1 mm.

**Figure 6:**
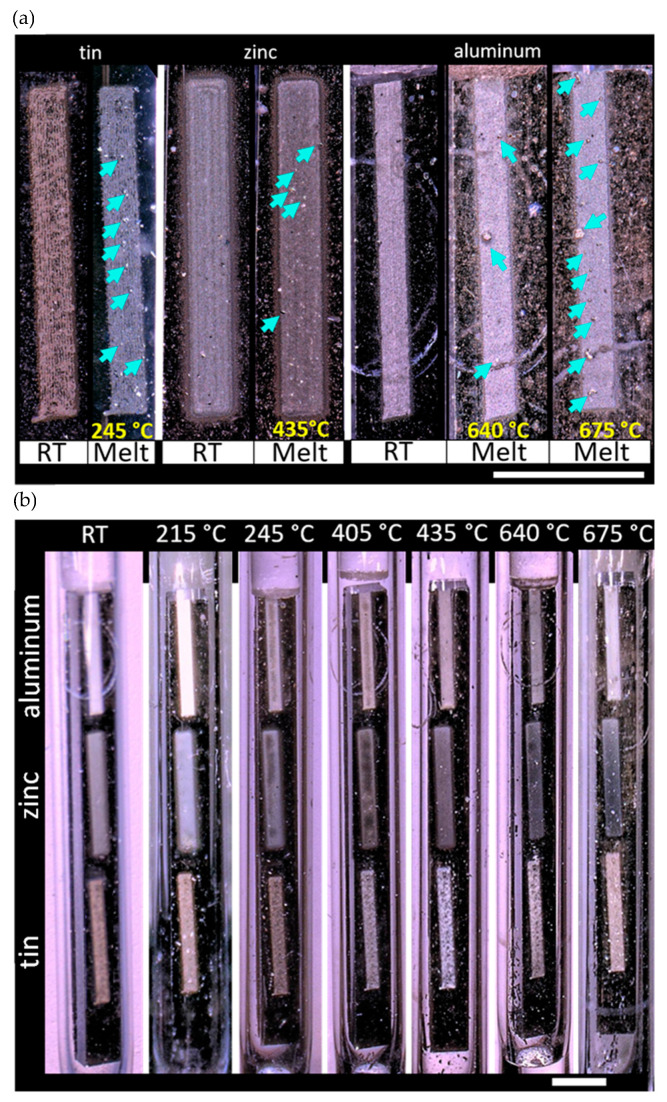
Micrographs of AM melt-wires in quartz capsule for furnace testing. (**a**) A magnified view of the temperature response indicating melting of individual melt wires of Sn, Zn, and Al, where the blue arrows indicate beading of the melt wire material due to melting. (**b**) Full view of quartz capsule with AM melt wires. Scale bars represent 1 mm.

For both melt-wire types, the furnace was brought up to the target temperature, and the melt wires were inserted so that they were positioned vertically during testing. To evaluate performance, each melt capsule was tested at a temperature slightly below and above the materials melting point, as determined by DSC. With that, tin was evaluated at 215 and 235 °C, zinc at 405 and 435 °C, and aluminum at 640 and 675 °C. Micrographs of the wires at room temperature (RT) and after melting are provided for both the standard and AM melt-wire capsules in Figure 5 and Figure 6, respectively. For the standard melt wire capsule, the aluminum wire (Figure 5a) had clearly melted prior to the expected melting point of 660 °C and testing at 675 °C was not performed.

During furnace testing of the AM melt wires, however, the melting behavior for each of the three different materials appeared to be consistent: bubbles or beads were observed to form after the metals were exposed to temperatures that were near or beyond their expected melting point (Figure 6). Interestingly, both the standard and AM aluminum melt wires displayed visual melting characteristics after having been exposed to a testing temperature of 640 °C, which is about 20 °C below the expected melting point determined through DSC.

Using the new encapsulation design, AM melt wire performance tests were completed with assemblies containing air and helium gas to evaluate the impact of the environment. Initial considerations towards melt wire containment did not entrap an inert atmosphere to ensure good melt wire performance because of the use of silver in initial evaluations, and oxidation was not of a significant concern [23]. However, most materials used to fabricate melt wires are prone to oxidation, and to support the use of these materials in an inert atmosphere is critical.

**Figure 7:**
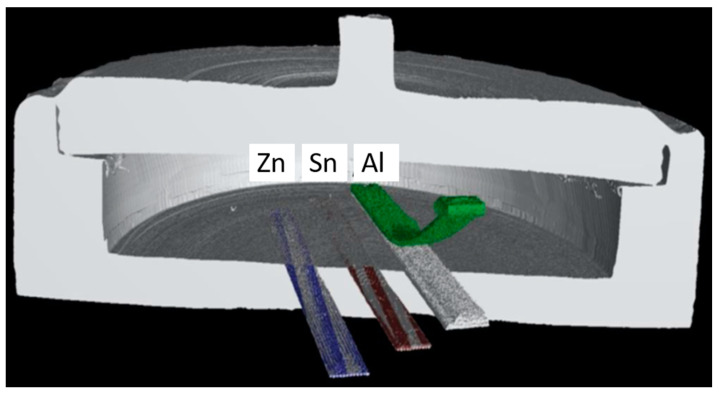
Cross section of a post furnace X-ray micro-computed tomography (XCT) image with zinc (blue), tin (maroon), superimposed over pre-furnaced image of a container sealed in air. Melt wires without color were taken before the furnace test. Melt wires with color were taken after exceeding the melting point of all three materials indicating no visible changes on two out of three wires when encapsulated in air.

Using SS316 as the encapsulation material eliminated the ability to use the traditional visual observation methods for evaluating the melt behavior of AM melt wires. To overcome this, XCT was used to produce detailed images of the melt wires before and after furnace tests. To demonstrate the need for an inert atmosphere, a melt wire assembly was assessed having an air encapsulation. A reference XCT image was collected before the melt wires were exposed to a temperature above their expected melting point. Those samples with an air encapsulation did not show distinguishable signs of melting even after exceeding expected melting temperatures more than 50 °C. However, the aluminum melt wire did exhibit some unexpected behavior when exposed to temperatures beyond its expected melting temperature as the line detached from the SS316 substrate and started bending upward. This uncharacteristic phenomenon can be observed in Figure 7, but this was only after reaching a temperature well in excess (greater than 50 °C) of its expected melting point of 660 °C.

An AM melt wire assembly having an inert encapsulation was then evaluated using the same method. A reference XCT image was collected before the melt wires were exposed to a temperature above all their expected melting points (680 °C). XCT images were then taken to evaluate the changes that should have occurred from melting. Contrast levels were adjusted for each melted material to provide the greatest detail for each image.

**Figure 8:**
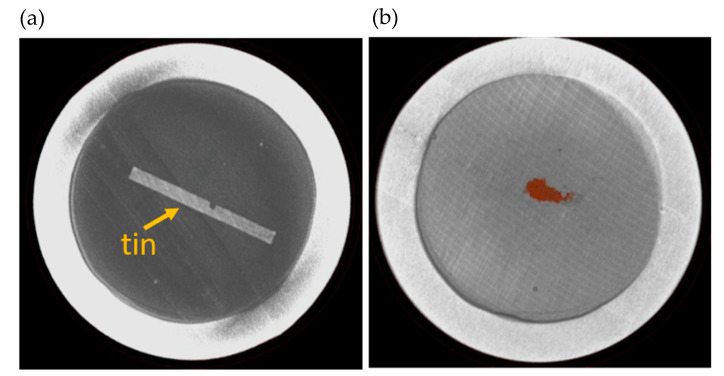
XCT images of AM tin melt wire. (**a**) Original XCT image of tin encapsulated in helium, and (**b**) post-furnace image of melted tin enhanced with color.

**Figure 9:**
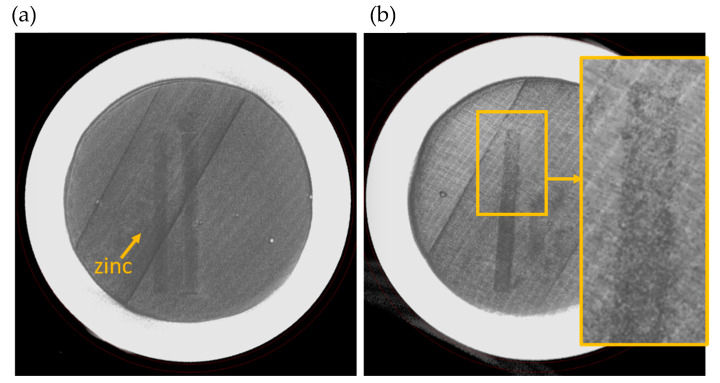
XCT images of AM zinc melt wire. (**a**) Original XCT image of zinc encapsulated in helium, (**b**) post-furnace image of zinc with enhanced image highlighting material separation.

The lowest melting point material was tin which had the most obvious changes compared to the other printed materials. From Figure 8 the tin collected in a pool near the center of the container. Zinc exhibited some subtle changes that could be identified to imply melting had occurred, which can be seen in Figure 9.

**Figure 10:**
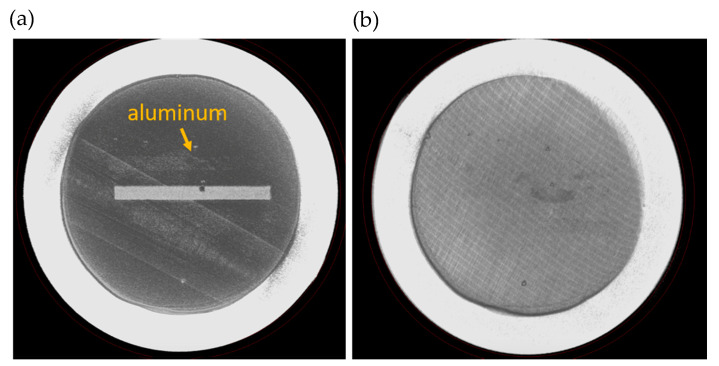
XCT images of AM aluminum melt wire. (**a**) Original XCT image of aluminum encapsulated in helium, (**b**) post-furnace image of melt wire capsule with the aluminum wire not discernable against the SS316 substrate.

When compared to the original XCT image it was observed that a section along the zinc wire showed separation. Finally, for the evaluation of aluminum (Figure 10), the printed line on the original XCT image was unclear, and in the post-furnace image the aluminum wire was even harder to identify. From these tests it was inconclusive as to whether aluminum had melted or was just unidentifiable against the encapsulation material.

## 4. Discussion

### 4.1. Powder Feedstock Characterization

The expected melting point derived from DSC analysis for each melt wire fabricated with AJP inks of tin, zinc or aluminum nanopowders are expected to be 228.3 ± 0.7 °C, 415 ± 1 °C, and 656.5 ± 0.3 °C, respectively. The melting points derived from DSC evaluation vary slightly from that of the standard values of 231.9 °C, 419.5 °C, and 660.3 °C for tin, zinc and aluminum, respectively (Table 1). These deviations are attributed to impurities within the feedstock materials, which results in the broadening of the melting point. For melt wire performance, consistent melt behavior of a material is far more important than obtaining a melting point that is close to the theoretical values. Understanding this, DSC measurements for each material are performed in triplicate to ensure that the melt behavior of the material is consistent.

### 4.2. AM and Standard Melt Wire Performance Evaluation

Normally, melt-wire materials within a capsule are chosen to provide information for a target location within a MTR that is not expected to exceed a certain temperature. For example, having a projected temperature of an experiment, material selection would begin with identifying a material having a melting point around 30 to 50 °C below the expected peak temperature. The next material selection would require the identification of a material having a melting point identical to or within 5 to 10 °C of the anticipated maximum temperature of the experiment, and the last material would include one having a melting point that exceeds projected experiment temperature by approximately 50 °C. Under normal experimental circumstances, this last material would not be expected to melt; however, this can provide information about unforeseen events during an experiment that may otherwise not be caught. Unlike a typical peak-temperature test, this material performance test was done to provide comparison information over a large range of melting temperatures to examine whether the melt-wire performance varied between standard and AM melt wires with materials having a wide range of melting temperatures. Melting evaluation for a material after an experiment can be challenging because not all melted materials have the same visual characteristics, as shown above. Figure 5a highlights the challenge of identifying optically whether melt wires have exceeded their melting temperature. It is very common to see that a melt wire has flowed to the bottom of the capsule when the capsules are in a vertical orientation, which is observed with zinc at temperatures well beyond its melting point (Figure 5b). On the other hand, some materials only exhibit softening and require identifying rounded edges where sharp features had existed in the non-melted wire, as seen with tin (Figure 5b). Other materials will draw together to form a sphere, or bubbles or beads sometimes form along abrupt edges or cracks while the bulk of the material appears unaffected due to surface tension, as seen with the aluminum melt wire (Figure 5b). Due to significant variations in visual melting characteristics, it is important to accurately identify what has occurred during prototype testing to recognize melting after an experiment.

**Figure 11:**
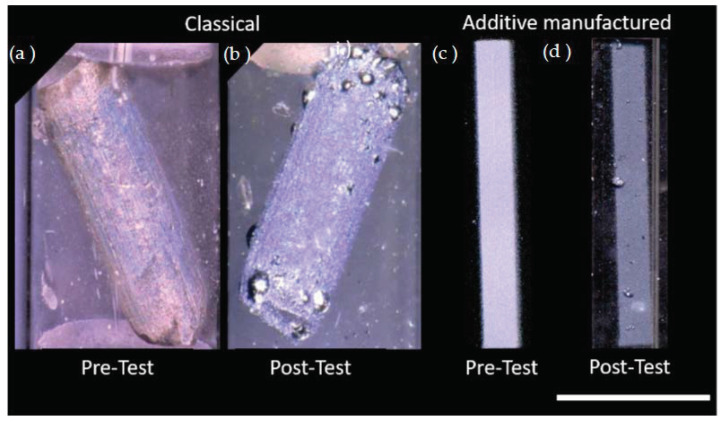
Comparison of melt behavior of classical and AM aluminum melt wires. Micrographs of the expected melt behavior of stock aluminum wire at room temperature (**a**) before DSC testing and (**b**) after DSC testing, for which the melting point was determined to be 660.5 °C. Micrographs of the observed melt behavior of AM melt wires (**c**) before and (**d**) after furnace testing. Scale bar represents 1 mm.

A phenomenon that is not as common, but is known to exist, includes materials reacting with one another during elevated temperatures causing vapor alloying or migration that results in material deposition onto another material or directly to the quartz tubing. This often results in a melting-point depression, as is observed with both forms of the aluminum wire. It is anticipated that this sort of interaction is the cause of the deviation from the expected melting point identified with DSC because this test is performed in an isolated and inert atmosphere. Furthermore, previous tests on the same stock material have resulted in an expected melting point (660.5 °C), and the melting characteristics from those tests are shown in Figure 11, with a comparison between the melt behavior of an aluminum classical (Figure 11a,b) and AM melt wire (Figure 11c,d) are highlighted.

Through the progression of micrographs in Figure 5 and Figure 6, material deposition is observed on the quartz tube, and this is more clearly visualized with the melt of zinc in Figure 5a, and material vaporization is further supported through Figure 6b. The zinc wire appears to fade while material buildup on the quartz tube demonstrates a direct relationship with test temperature. Through these observations, it is concluded that, during the testing process, a vapor alloying-like process has occurred, which is responsible for the melting point depression of the standard and AM aluminum melt wires.

The ability to further miniaturize the melt wire package was introduced with the use of AM methods as it provides a way to deposit melt wire materials directly onto a substrate, and this allowed for an exploration into different encapsulation designs that would not otherwise be possible. The use of different melt wire materials for peak temperature monitoring also introduces the consideration that different materials have different melting characteristics, and it is important to identify features before using them within an irradiation experiment. These changes can include but are not limited to rounded edges where sharp features had previously existed, the formation of a sphere of the entire melted line, or the development of bubbles or beads while the bulk of the material appears unaffected. Characterization of AM melt wires requires an assessment of each material’s melt behavior by identifying changes between XCT images before and after exposure to melting temperatures. For this work, the evaluation method used for classical melt wires could not be used due to the opaque encapsulation material. Therefore, XCT was chosen because it is a capability that is available for PIE and it enables imaging of the melt wire materials through opaque encapsulation.

With the use of the new encapsulation design, it was observed that melt identification could be made for tin and zinc after having exposed the melt wire capsule (with inert environment), but not for aluminum due to the low contrast between the aluminum melt wire and the SS316 substrate. Furthermore, the melt behavior of the same materials on a SiO_2_/Si substrate and a stainless steel substrate showed inconsistencies, which highlight the need to characterize the melt behavior of AM melt wires with each substrate they are deposited on. With these results, future work will require the exploration of additional encapsulation materials and/or melt wire materials focused towards selecting encapsulation and melt materials that have high contrast for the XCT imaging process.

AM methods provide a novel approach towards device miniaturization. While the melt wires themselves may be miniaturized, future work must focus on methods to miniaturize the entire melt wire package to include the melt wire encapsulation. To elaborate, the use of quartz as the transparent encapsulation material limits the ability to miniaturize a melt wire package, and materials that support these efforts are generally opaque and would require the development of welding and sealing methods that would provide both protection and a controlled environment to the printed melt wires. Furthermore, the use of different encapsulation methods and materials would, in turn, require higher-resolution microscopy or the incorporation of methods, such as XCT, if opaque encapsulation materials are used.

Melting evaluation for a material after an experiment can be challenging because not all melted materials have the same visual characteristics, as seen in Figure 5 and Figure 6. Additionally, this work demonstrates that for AM melt wires, the choice of substrate will play a role in melt behavior, as different melting characteristics were observed between the SiO_2_/Si substrate and SS316. It is very common to see that a melt wire has flowed to the bottom of the capsule if the capsules are in a vertical orientation, which is observed with zinc at temperatures well beyond its melting point (Figure 5b). Finally, the use of higher-resolution microscopy methods will be required to better visualize melt-wire features and melting characteristics of AM melt wires. This introduces the need to consider potential issues with X-ray contrast in material selection between substrate and melt wire material. Ultimately, the incorporation of AM melt wires would supply a complimentary peak test-temperature determination, and a better melt-evaluation technique for AM melt wires.

## 5. Conclusions

AM methods, such as AJP, possess significant potential for the fabrication of advanced sensors and instrumentation. This is especially true for instances where miniaturization of sensors is required due to space limitations within an experiment. To demonstrate the feasibility of incorporating AM techniques for the fabrication of advanced nuclear in-pile passive temperature sensors, a comparison between standard and AM melt wires of tin, aluminum and zinc was completed. Through DSC and furnace testing, it was determined that the performance of the AM melt-wire capsule was consistent with that of the standard melt-wire capsule.

## Figures and Tables

**Table 1:** DSC melting point results.

Material	Theoretical Melting Point	Melting Point Derived from DSC Powder Feedstock	Meling Point Derived from DSC Bulk Wire
Tin	231.9 °C	228.3 ± 0.7 °C	231.8 °C
Zinc	419.5 °C	415 ± 1 °C	419.3 °C
Aluminum	660.3 °C	656.5 ± 0.3 °C	660.5 °C

## Data Availability

Not applicable.
